# Supplementary material for: Analysis of cause-effect inference by comparing regression errors
Source: PeerJ Comput Sci. 2019 Jan 21;5:e169. doi: 10.7717/peerj-cs.169 (PMC7924496; doi:10.7717/peerj-cs.169)
Supplement: Supplemental Information 1 — An overview of all results and parameters. [file peerj-cs-05-169-s001.pdf]

1 RESULTS OF STANDARDIZED DATA

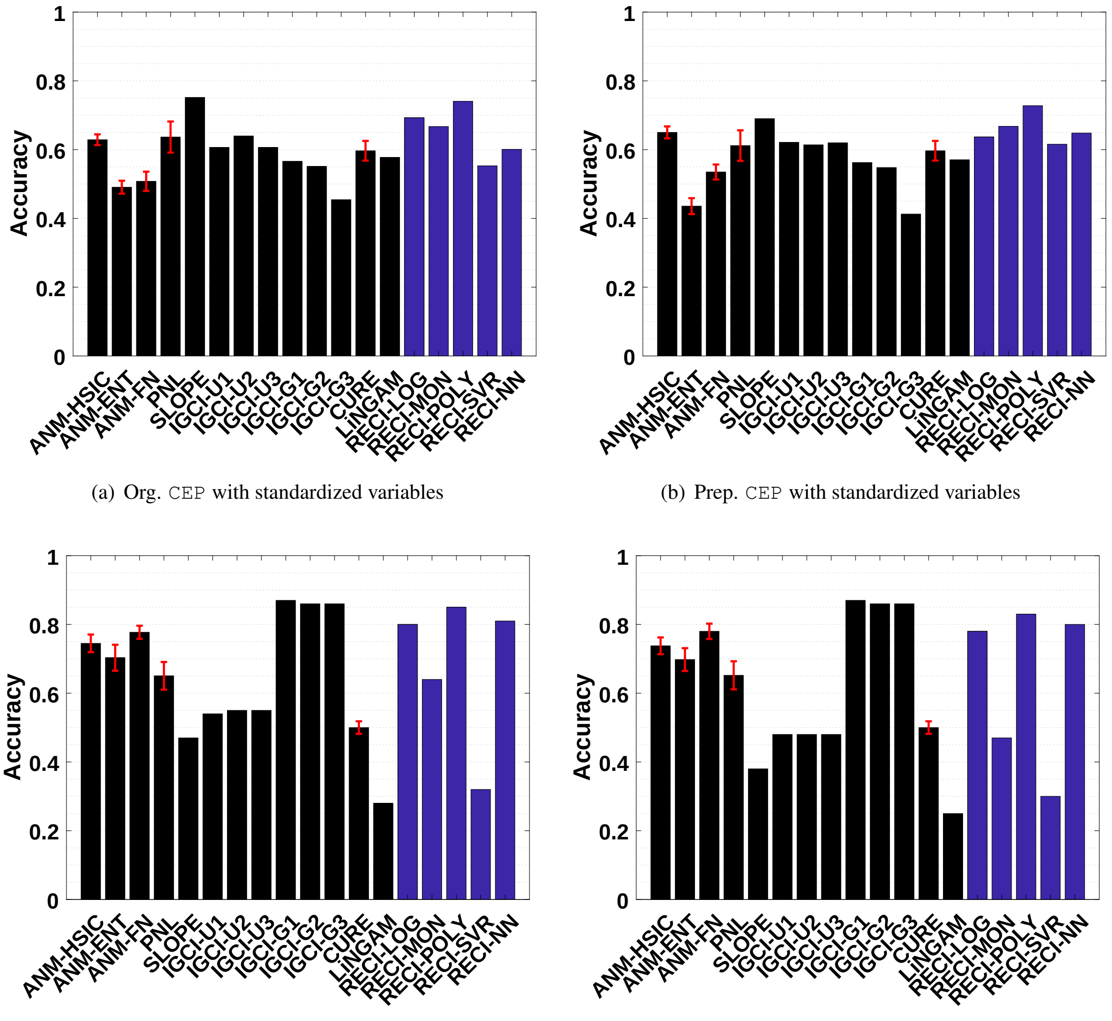

(a) Org. CEP with standardized variables

(b) Prep. CEP with standardized variables

(c) Org. SIM-G with standardized variables

(d) Prep. SIM-G with standardized variables

Figure 1. Evaluation results of all methods in the real-world CEP and SIM-G data sets. Here, a standardization instead of a normalization was used for the scaling in RECI. The figure on the left side shows the results of the evaluations in the original data and on the right side the results in the preprocessed versions where low-density points were removed.

2 TABLES

Table 1. The performances of all used classes of regression functions for RECI in the artificial and real-world benchmark data sets when averaging the MSE over all runs.

	`CEP`	Prep. `CEP`	`SIM`	Prep. `SIM`	`SIM-c`	Prep. `SIM-c`	`SIM-ln`	Prep. `SIM-ln`	`SIM-G`	Prep. `SIM-G`
LOG	68.93%	70.02%	**61**%	**71**%	**70**%	**74**%	72%	**80**%	**72**%	**66**%
ax^2+c	**72.13**%	**75.41**%	43%	52%	57%	63%	44%	47%	44%	33%
ax^3+c	69.07%	72.67%	45%	55%	54%	60%	45%	47%	43%	34%
ax^4+c	68.84%	72.67%	42%	53%	56%	58%	45%	47%	43%	37%
ax^5+c	69.92%	71.99%	43%	54%	60%	58%	44%	44%	45%	35%
ax^6+c	68.84%	70.16%	45%	55%	61%	63%	40%	41%	45%	36%
ax^7+c	67.75%	68.93%	46%	54%	62%	64%	42%	40%	47%	35%
ax^8+c	67.61%	67.42%	46%	55%	62%	60%	38%	39%	44%	34%
ax^9+c	66.56%	69.93%	46%	53%	63%	62%	37%	39%	45%	36%
$\sum_{i=0}^{1} a_i x^i$	67.47%	66.51%	46%	54%	51%	58%	45%	43%	41%	26%
$\sum_{i=0}^{2} a_i x^i$	67.7%	64.26%	53%	58%	65%	68%	69%	70%	54%	49%
$\sum_{i=0}^{3} a_i x^i$	68.61%	64.49%	57%	59%	67%	71%	78%	76%	57%	52%
$\sum_{i=0}^{4} a_i x^i$	66.63%	64.26%	57%	61%	68%	72%	80%	77%	60%	55%
$\sum_{i=0}^{5} a_i x^i$	66.10%	66.54%	55%	59%	68%	71%	80%	75%	60%	53%
$\sum_{i=0}^{6} a_i x^i$	66.08%	67%	52%	57%	68%	70%	**82**%	76%	59%	54%
$\sum_{i=0}^{7} a_i x^i$	66.36%	65.59%	57%	57%	68%	72%	81%	74%	60%	54%
$\sum_{i=0}^{8} a_i x^i$	65.55%	64.26%	56%	59%	68%	69%	**82**%	75%	60%	54%
$\sum_{i=0}^{9} a_i x^i$	65.28%	63.34%	55%	61%	68%	69%	78%	71%	59%	55%
SVR	68.84%	66.51%	48%	54%	52%	59%	45%	43%	40%	26%
NN 2	63.82%	63.34%	54%	57%	68%	72%	81%	79%	60%	56%
NN 5	68.48%	65.85%	56%	55%	67%	70%	79%	76%	61%	56%
NN 10	64.82%	66.63%	54%	56%	68%	69%	80%	70%	62%	56%
NN 20	67.81%	70.53%	54%	58%	66%	70%	81%	72%	60%	57%
NN 2-4	64.28%	64.12%	55%	58%	68%	68%	78%	76%	60%	54%
NN 4-8	67.47%	65.85%	52%	56%	68%	68%	77%	71%	61%	55%

Table 2. The performances of all used classes of regression functions for RECI in the artificial and real-world benchmark data sets when not averaging the MSE.

	`CEP`	Prep. `CEP`	`SIM`	Prep. `SIM`	`SIM-c`	Prep. `SIM-c`	`SIM-ln`	Prep. `SIM-ln`	`SIM-G`	Prep. `SIM-G`
LOG	63.66% ± 3.48	64.34% ± 3.57	57.62% ± 3.57	60.83% ± 3.83	65.02% ± 3.82	67.52% ± 3.3	71.74% ± 4.02	72.71% ± 3.49	**67.37**% ± 4.15	**61.95**% ± 4.35
ax^2+c	**70.73**% ± 1.55	**72.90**% ± 0.00	43.84% ± 1.17	52% ± 0.00	52.74% ± 1.07	60% ± 0.00	44.45% ± 1.29	47% ± 0.00	43.74% ± 0.91	33.56% ± 1.78
ax^3+c	70.12% ± 1.71	72.67% ± 0.00	45.47% ± 1.78	54% ± 0.00	53.37% ± 0.94	58% ± 0.00	45.08% ± 1.28	47.63% ± 1.13	42.77% ± 1.72	34.80% ± 1.69
ax^4+c	69.39% ± 1.39	72.67% ± 0.00	43.92% ± 0.01	53% ± 0.00	56.42% ± 1.44	58.52% ± 1.87	45% ± 0.00	47% ± 0.00	44.11% ± 1.62	34.54% ± 1.78
ax^5+c	69.20% ± 1.82	70.5% ± 2.18	43.66% ± 0.02	54% ± 0.00	58.11% ± 1.78	58.54% ± 1.17	43.76% ± 1.66	43.22% ± 0.02	44.59% ± 1.99	35.47% ± 2.39
ax^6+c	68.84% ± 0.00	69.63% ± 2.47	43.68% ± 0.02	55% ± 0.00	59% ± 0.00	62.99% ± 0.1	41.42% ± 1.83	43.51% ± 0.41	44% ± 0.00	36.78% ± 2.11
ax^7+c	67.35% ± 2.54	68.01% ± 2.61	44.07% ± 0.12	53.92% ± 1.73	60% ± 0.00	64% ± 0.00	41.76% ± 2.31	42.17% ± 0.11	44% ± 0.00	35.25% ± 2.44
ax^8+c	67.27% ± 2.31	67.27% ± 2.44	44.12% ± 1.24	54.89% ± 1.8	60.27% ± 1.58	60.18% ± 1.01	39.17% ± 0.57	41.85% ± 0.14	43.27% ± 2.26	34% ± 1.94
ax^9+c	66.78% ± 2.25	66.67% ± 2.77	44.47% ± 2.12	54.27% ± 1.84	61% ± 0.00	62% ± 0.00	37.69% ± 0.00	41.19% ± 0.08	43% ± 0.14	34.3% ± 2.83
$\sum_{i=0}^{1} a_i x^i$	67.54% ± 0.00	66.28% ± 0.00	45.77% ± 1.54	54.37% ± 1.47	51% ± 0.00	58.24% ± 1.62	45% ± 0.00	43% ± 0.00	41% ± 0.00	24.13% ± 1.05
$\sum_{i=0}^{2} a_i x^i$	66.99% ± 0.00	66% ± 2.64	53.53% ± 1.86	59.54% ± 2.11	65% ± 0.00	68% ± 0.00	69% ± 0.00	71.47% ± 1.04	54.08% ± 1.68	49% ± 0.00
$\sum_{i=0}^{3} a_i x^i$	67.24% ± 0.00	65.4% ± 1.97	57.67% ± 2.4	59.91% ± 2.54	**67**% ± 0.00	**70**% ± 0.00	75% ± 0.00	75.54% ± 1.22	57.26% ± 1.95	52.01% ± 2.31
$\sum_{i=0}^{4} a_i x^i$	63.87% ± 2.78	66.81% ± 0.00	57.47% ± 2.37	60.72% ± 2.59	68% ± 0.00	**70**% ± 0.00	77% ± 0.00	75.63% ± 1.45	59.35% ± 2.11	54% ± 0.00
$\sum_{i=0}^{5} a_i x^i$	65.19% ± 0.00	66.54% ± 0.00	56.59% ± 2.48	58.92% ± 2.47	**67**% ± 0.00	**70**% ± 0.00	78% ± 0.00	74.43% ± 1.46	59% ± 0.00	53.06% ± 2.37
$\sum_{i=0}^{6} a_i x^i$	64.76% ± 2.91	67% ± 0.00	54.17% ± 1.98	**61.81**% ± 2.72	**67**% ± 0.00	69% ± 0.00	80% ± 0.00	73% ± 0.00	58.51% ± 2.19	53.82% ± 2.41
$\sum_{i=0}^{7} a_i x^i$	65.08% ± 0.00	66.36% ± 0.00	55.39% ± 2.05	58.99% ± 2.54	**67**% ± 0.00	69% ± 0.00	79% ± 0.00	72% ± 0.00	58.94% ± 2.87	53.01% ± 2.89
$\sum_{i=0}^{8} a_i x^i$	65.07% ± 0.00	66.24% ± 0.00	55.19% ± 2.23	58.18% ± 2.94	62.06% ± 2.86	68% ± 0.00	79% ± 0.00	72% ± 0.00	58.47% ± 2.14	53.47% ± 2.94
$\sum_{i=0}^{9} a_i x^i$	64.94% ± 0.00	67.21% ± 0.00	54.72% ± 2.59	61.15% ± 2.41	**67**% ± 0.00	69% ± 0.00	77% ± 0.00	72% ± 0.00	57.57% ± 2.61	53% ± 0.00
SVR	66% ± 0.38	64.67% ± 2.73	45.47% ± 1.49	54.33% ± 2.06	52% ± 0.00	58% ± 0.00	41.37% ± 2.1	43% ± 0.00	40% ± 0.00	25.69% ± 1.88
NN 2	63.5% ± 1.94	64.03% ± 1.57	54.41% ± 2.54	58.17% ± 2.21	66.54% ± 1.78	68.36% ± 2	**80.97**% ± 1.48	**76.97**% ± 2.31	59.65% ± 2.43	54.93% ± 2.89
NN 5	63.92% ± 2.06	65.69% ± 1.52	56.69% ± 2.56	57.08% ± 2.54	66.49% ± 0.97	68.26% ± 2.28	79.72% ± 1.74	74.58% ± 3.17	60.26% ± 2.53	54.67% ± 3.11
NN 10	64.83% ± 2.44	66.07% ± 1.46	55.16% ± 2.36	58.07% ± 2.95	66.82% ± 1.1	68.71% ± 2.26	76.99% ± 2.41	73.47% ± 3.29	59.48% ± 2.38	54.49% ± 2.85
NN 20	64.9% ± 3.02	66.87% ± 2.19	**57.65**% ± 2.26	59.72% ± 3.23	66.36% ± 2.78	69.46% ± 2.42	76.36% ± 2.84	73.10% ± 2.83	58.98% ± 2.9	54.83% ± 3.21
NN 2-4	63.26% ± 1.87	65.33% ± 1.61	56.72% ± 2.5	57.93% ± 2.56	66.52% ± 1.02	66.89% ± 2.44	80.25% ± 1.81	74.04% ± 2.16	59.98% ± 2.46	54.26% ± 2.83
NN 4-8	64.33% ± 2.23	66.05% ± 1.44	54.96% ± 2.73	57.82% ± 2.91	66.48% ± 1.77	68.5% ± 2.59	78.71% ± 2.01	73.12% ± 2.41	59.76% ± 2.51	55.44% ± 2.61

Table 3. The performances of all used classes of regression functions for RECI in the standardized `CEP` and `SIM-G` data sets when averaging the MSE over all runs.

	Standardized `CEP`	Standardized Prep. `CEP`	Standardized `SIM-G`	Standardized Prep. `SIM-G`
LOG	69.29%	66.87%	80%	78%
ax^2+c	63.57%	66.53%	24%	21%
ax^3+c	61.99%	57.83%	60%	47%
ax^4+c	65.44%	66.79%	29%	18%
ax^5+c	64.61%	62.71%	58%	43%
ax^6+c	65.65%	65.72%	31%	19%
ax^7+c	64.49%	64.26%	58%	47%
ax^8+c	66.72%	65.76%	36%	26%
ax^9+c	65.86%	65.84%	64%	44%
$\sum_{i=0}^{1} a_i x^i$	60.83%	61.25%	31%	30%
$\sum_{i=0}^{2} a_i x^i$	**74.07**%	**72.79**%	83%	79%
$\sum_{i=0}^{3} a_i x^i$	67.47%	66.01%	83%	80%
$\sum_{i=0}^{4} a_i x^i$	67.28%	66.07%	**85**%	**83**%
$\sum_{i=0}^{5} a_i x^i$	62.4%	66.95%	80%	82%
$\sum_{i=0}^{6} a_i x^i$	67.97%	64%	80%	81%
$\sum_{i=0}^{7} a_i x^i$	64.43%	63.06%	78%	77%
$\sum_{i=0}^{8} a_i x^i$	67.87%	65.70%	80%	82%
$\sum_{i=0}^{9} a_i x^i$	68.89%	68.31%	81%	81%
SVR	55.30%	61.58%	32%	30%
NN 2	55.19%	64.82%	80%	78%
NN 5	56.85%	61.35%	81%	76%
NN 10	60.57%	58.90%	80%	80%
NN 20	56.75%	60.89%	79%	79%
NN 2-4	60.09%	57.78%	72%	77%
NN 4-8	59.13%	59.21%	80%	80%

Table 4. The performances of all used classes of regression functions for RECI in the standardized `CEP` and `SIM-G` data sets when not averaging the MSE.

	Standardized `CEP`	Standardized Prep. `CEP`	Standardized `SIM-G`	Standardized Prep. `SIM-G`
LOG	53.86% ± 5.33	54.18% ± 6.12	76.67% ± 3.26	76.21% ± 3.13
$ax^2 + c$	62.41% ± 0.00	58.88% ± 0.00	34.94% ± 4.51	26.23% ± 3.86
$ax^3 + c$	61.99% ± 0.00	56.67% ± 0.81	54.42% ± 3.53	47.91% ± 4.12
$ax^4 + c$	65.44% ± 0.00	59.68% ± 0.00	32.44% ± 3.18	26.23% ± 3.45
$ax^5 + c$	56.80% ± 0.00	58.79% ± 0.02	54.65% ± 3.87	45.74% ± 4.03
$ax^6 + c$	65.65% ± 0.00	60.43% ± 0.00	36.24% ± 2.97	35.60% ± 3.80
$ax^7 + c$	60.53% ± 0.00	57.39% ± 0.00	51.44% ± 3.63	44.65% ± 4.10
$ax^8 + c$	**65.73**% ± 0.63	62.91% ± 0.00	38.8% ± 3.4	31.20% ± 4.89
$ax^9 + c$	63.72% ± 0.92	58.79% ± 0.00	53% ± 0.00	50% ± 0.00
$\sum_{i=0}^{1} a_i x^i$	56.05% ± 5.73	57.78% ± 6.10	43.45% ± 0.00	43.87% ± 4.04
$\sum_{i=0}^{2} a_i x^i$	60.03% ± 5.66	61.73% ± 0.00	**83**% ± 0.00	77% ± 0.00
$\sum_{i=0}^{3} a_i x^i$	60.52% ± 0.00	59.14% ± 4.47	**83**% ± 0.00	79% ± 0.00
$\sum_{i=0}^{4} a_i x^i$	62.71% ± 4.45	61.14% ± 4.65	**83**% ± 0.00	**83**% ± 0.00
$\sum_{i=0}^{5} a_i x^i$	61.08% ± 4.14	62.07% ± 4.54	79.27% ± 1.34	81% ± 0.00
$\sum_{i=0}^{6} a_i x^i$	61.93% ± 4.87	61.73% ± 4.47	78.39% ± 1.44	80% ± 0.00
$\sum_{i=0}^{7} a_i x^i$	61.76% ± 4.07	61.23% ± 4.79	77.85% ± 1.98	76.59% ± 3.17
$\sum_{i=0}^{8} a_i x^i$	62.37% ± 5.10	**63.35**% ± 4.20	77.8% ± 1.78	82% ± 0.00
$\sum_{i=0}^{9} a_i x^i$	63.70% ± 4.53	63.27% ± 4.16	76.83% ± 1.71	81% ± 0.00
SVR	55.12% ± 5.50	57.15% ± 5.49	44.53% ± 4.23	42.69% ± 2.01
NN 2	55.89% ± 5.30	57.12% ± 5.70	80.07% ± 2.38	80.04% ± 2.74
NN 5	55.61% ± 5.87	57.31% ± 6.08	78.10% ± 2.23	77.29% ± 2.35
NN 10	56.63% ± 5.37	57.08% ± 3.52	76.68% ± 2.41	77.10% ± 2.50
NN 20	55.93% ± 6.55	58.07% ± 5.76	75.45% ± 2.76	75.78% ± 2.94
NN 2-4	56.26% ± 6.47	56.27% ± 6.03	78.71% ± 2.69	78.26% ± 2.62
NN 4-8	56.57% ± 3.70	57.65% ± 3.47	77.04% ± 2.19	77.60% ± 2.40

Table 5. All performances of ANM, PNL, SLOPE, IGCI, CURE and LiNGAM.

	CEP	Prep. CEP	SIM	Prep. SIM	SIM-c	Prep. SIM-c	SIM-ln	Prep. SIM-ln	SIM-G	Prep. SIM-G
ANM-HSIC	62.89% ± 1.56	65.01% ± 1.74	**75.54**% ± 2.14	**73.33**% ± 2.08	**80.54**% ± 2.14	**77.93**% ± 2.02	76.60% ± 2.53	70.98% ± 2.40	74.49% ± 2.57	73.78% ± 2.44
ANM-ENT	49.07% ± 1.88	43.57% ± 2.31	72.91% ± 3.09	71.51% ± 3.35	76.36% ± 3.14	74.95% ± 3.57	78.88% ± 2.63	73.93% ± 2.88	70.32% ± 3.77	69.78% ± 3.32
ANM-FN	50.80% ± 2.80	53.50% ± 2.18	57.50% ± 1.60	51.94% ± 1.92	61.15% ± 1.99	59.57% ± 1.93	**85.31**% ± 1.36	**80.03**% ± 1.31	77.72% ± 1.90	78% ± 2.23
PNL	63.66% ± 1.65	61.18% ± 1.62	66.33% ± 4.25	65.53% ± 3.99	68.85% ± 3.35	67.71% ± 3.16	52.21% ± 4.02	53.39% ± 4.00	65.06% ± 4.02	65.20% ± 4.08
SLOPE	**75.19**% ± 0.00	**69.02**% ± 0.00	44% ± 0.00	54% ± 0.00	55% ± 0.00	61% ± 0.00	47% ± 0.00	46% ± 0.00	47% ± 0.00	38% ± 0.00
$IGCI_{U,1}$	60.66% ± 0.00	62.13% ± 0.00	36% ± 0.00	39% ± 0.00	46% ± 0.00	47% ± 0.00	51% ± 0.00	57% ± 0.00	54% ± 0.00	48% ± 0.00
$IGCI_{U,2}$	63.97% ± 0.00	61.40% ± 0.00	41% ± 0.00	42% ± 0.00	50% ± 0.00	52% ± 0.00	52% ± 0.00	57% ± 0.00	55% ± 0.00	48% ± 0.00
$IGCI_{U,3}$	60.67% ± 0.00	61.99% ± 0.00	41% ± 0.00	42% ± 0.00	50% ± 0.00	52% ± 0.00	52% ± 0.00	57% ± 0.00	55% ± 0.00	48% ± 0.00
$IGCI_{G,1}$	56.62% ± 0.00	56.26% ± 0.00	36% ± 0.00	36% ± 0.00	42% ± 0.00	41% ± 0.00	59% ± 0.00	57% ± 0.00	**87**% ± 0.00	**87**% ± 0.00
$IGCI_{G,2}$	55.15% ± 0.00	54.79% ± 0.00	37% ± 0.00	38% ± 0.00	46% ± 0.00	45% ± 0.00	61% ± 0.00	60% ± 0.00	86% ± 0.00	86% ± 0.00
$IGCI_{G,3}$	45.45% ± 0.00	41.26% ± 0.00	37% ± 0.00	38% ± 0.00	46% ± 0.00	45% ± 0.00	61% ± 0.00	60% ± 0.00	86% ± 0.00	86% ± 0.00
CURE	59.67% ± 2.86	59.67% ± 2.86	59.62% ± 3.30	59.62% ± 3.30	68.60% ± 3.68	68.60% ± 3.68	55.58% ± 1.82	55.58% ± 1.82	47.46% ± 0.96	47.46% ± 0.96
LiNGAM	57.76% ± 0.00	57.06% ± 0.00	43% ± 0.00	49% ± 0.00	47% ± 0.00	52% ± 0.00	24% ± 0.00	25% ± 0.00	28% ± 0.00	25% ± 0.00

Table 6. The best performing parameters for each causal inference method in the artificial data sets `Linear`, `Invertible` and `Non-invertible`.

Data set	ANM estimator	IGCI configuration	RECI regression model
Linear	FN	IGCI-U1	MON: $ax^9 + c$
Invertible	HSIC	IGCI-U2	NN 4-8
Non-invertible	HSIC	IGCI-U1	POLY: $\sum_{i=0}^{4} a_i x^i$

Table 7. An overview of all data sets with the corresponding number of cause-effect pairs and data samples.

**Dataset**	**Number of Cause-Effect Pairs**	**Number of Samples per Pair**
`SIM`	100	1000
`SIM-c`	100	1000
`SIM-ln`	100	1000
`SIM-G`	100	1000
`Linear`	1100	500
`Invertible`	4100	500
`Non-invertible`	1100	500

**`CEP` Pair**	1	2	3	4	5	6	7	8	9	10
**Number of Samples**	349	349	349	349	4177	4177	4177	4177	4177	4177

11	12	13	14	15	16	17	18	19	20	21	22	23	24
4177	5000	392	392	392	392	5000	314	194	349	349	450	450	451

25	26	27	28	29	30	31	32	33	34	35	36	37	38
1030	1030	1030	1030	1030	1030	1030	1030	345	345	345	345	345	757

39	40	41	42	43	44	45	46	47	48	49	50	51	52
394	733	763	9162	10369	10369	10369	10369	254	168	365	365	365	192

53	54	55	56	57	58	59	60	61	62	63	64	65	66
192	192	192	192	192	192	192	162	1331	1331	1331	498	16382	4499

67	68	69	70	71	72	73	74	75	76	77	78	79	80
1632	5084	194	205	347	8401	721	721	721	365	365	365	3102	994

81	82	83	84	85	86	87	88	89	90	91	92	93	94
666	7753	261	131	1261	149	150	432	9504	9504	9504	202	94	2287

95	96	97	98	99	100
2287	300	109	109	109	114

3 PLOTS

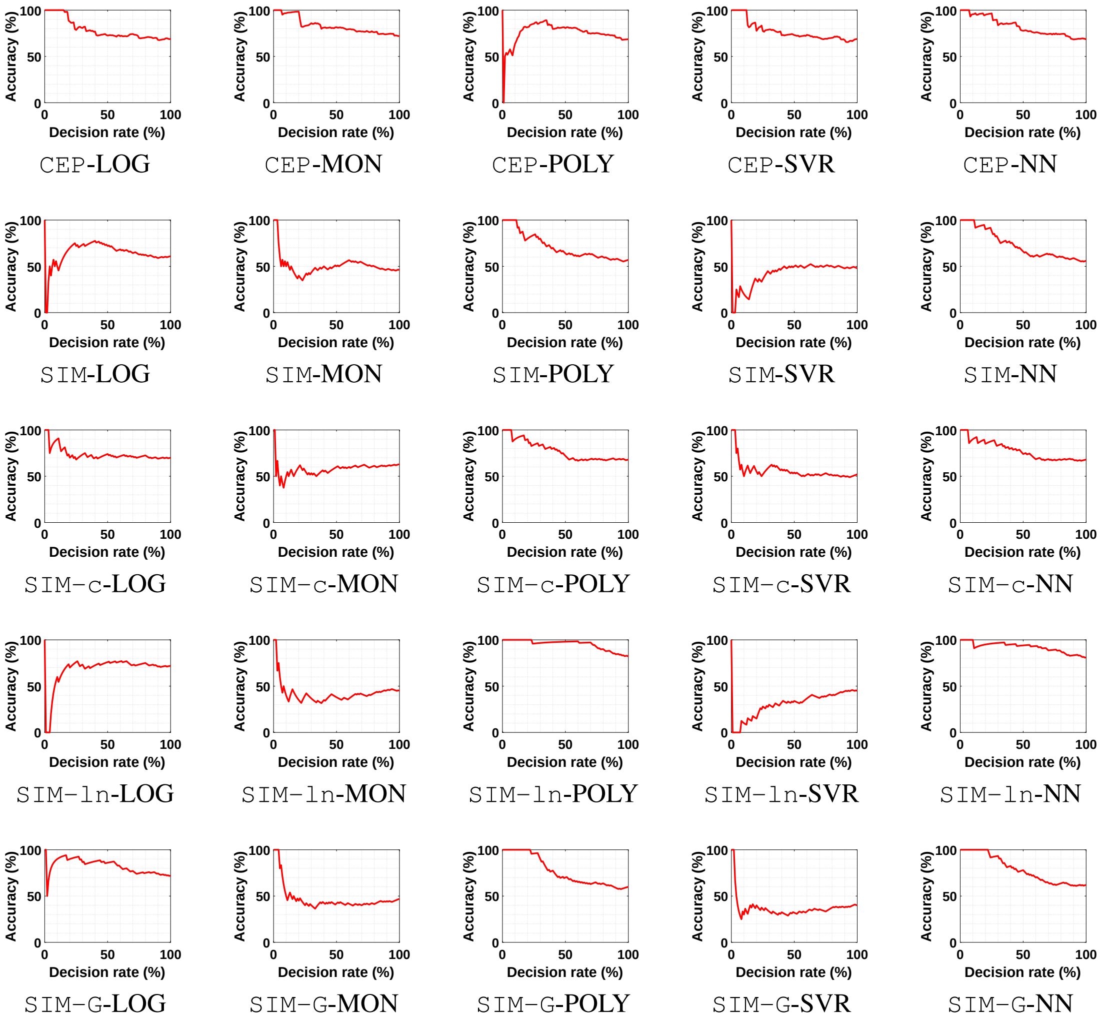

Figure 2. The performance of RECI in the original data sets if a certain decisions rate is forced. Here, the decisions are ranked according to the confidence measure defined in (21).

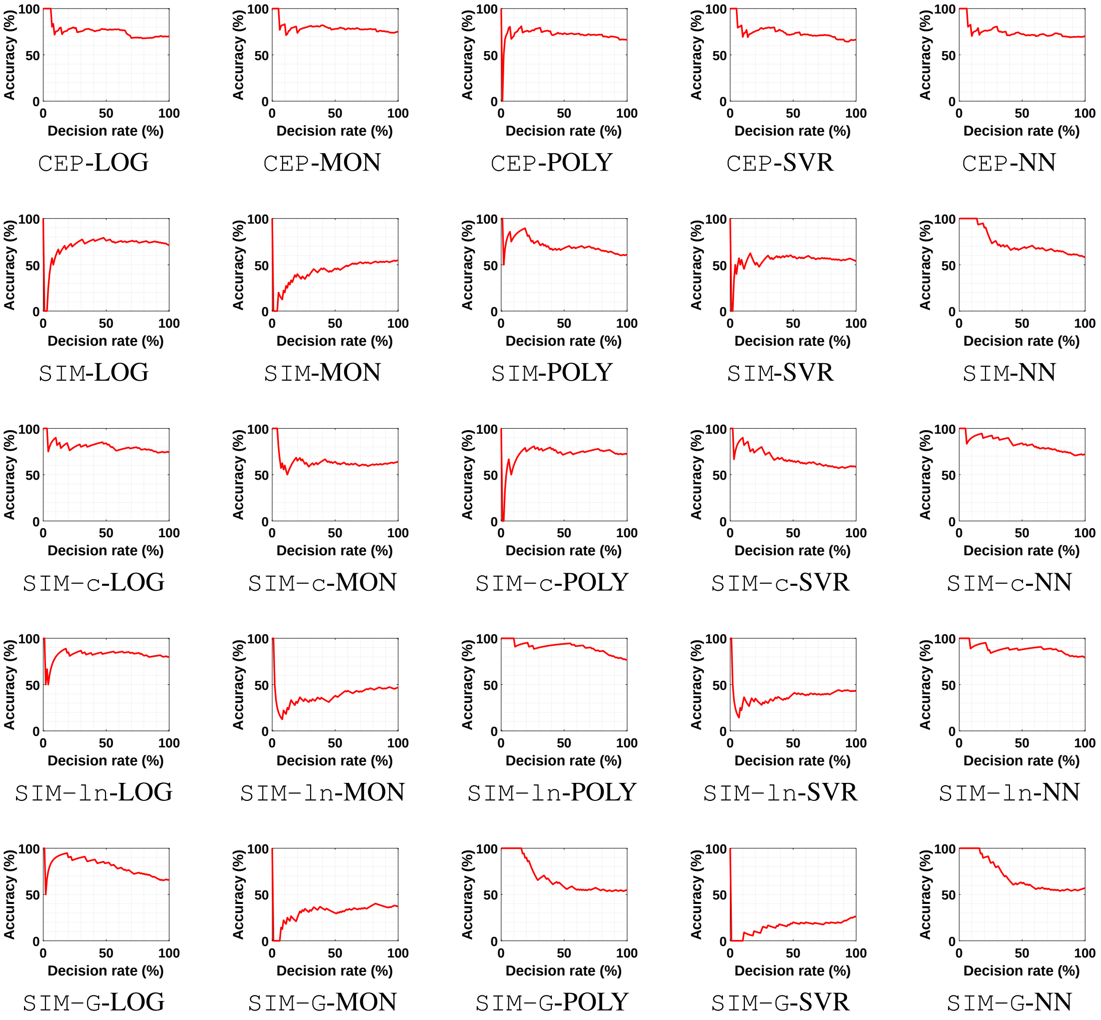

Figure 3. The performance of RECI in the preprocessed data sets if a certain decisions rate is forced. Here, the decisions are ranked according to the confidence measure defined in (21).

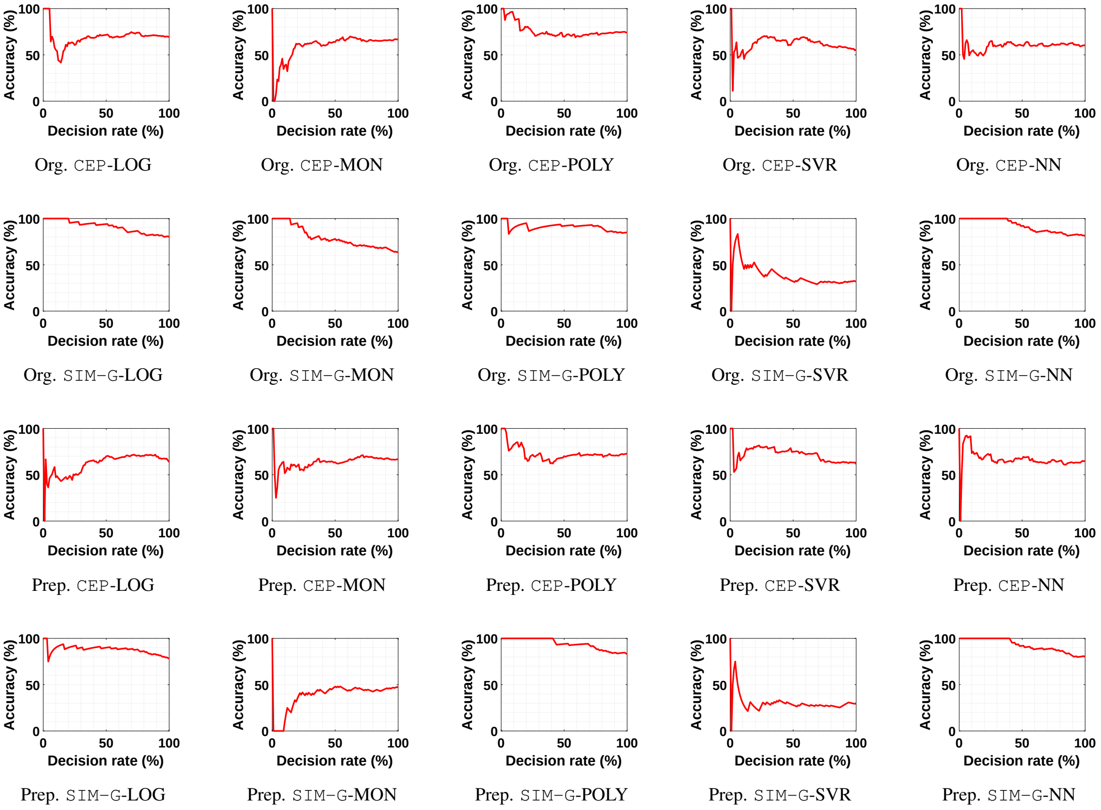

Figure 4. The performance of RECI in the standardized data sets if a certain decisions rate is forced. Here, the decisions are ranked according to the confidence measure defined in (21).
